# Roles of Phosphorus Sources in Microbial Community Assembly for the Removal of Organic Matters and Ammonia in Activated Sludge

**DOI:** 10.3389/fmicb.2019.01023

**Published:** 2019-05-16

**Authors:** Lei Zheng, Mengli Ren, En Xie, Aizhong Ding, Yan Liu, Songqiang Deng, Dayi Zhang

**Affiliations:** ^1^College of Water Science, Beijing Normal University, Beijing, China; ^2^College of Water Resources and Civil Engineering, China Agricultural University, Beijing, China; ^3^Chinese Research Academy of Environmental Sciences, Beijing, China; ^4^Research Institute for Environmental Innovation (Tsinghua-Suzhou), Suzhou, China; ^5^School of Environment, Tsinghua University, Beijing, China

**Keywords:** phosphorus, activated sludge, alkaline phosphatase, microbial community structure, AKP-associated gene

## Abstract

Various phosphorus sources are utilized by microbes in WWTPs, eventually affecting microbial assembly and functions. This study identified the effects of phosphorus source on microbial communities and functions in the activated sludge. By cultivation with 59 phosphorus sources, including inorganic phosphates (IP), nucleoside-monophosphates (NMP), cyclic-nucleoside-monophosphates (cNMP), and other organophosphates (OP), we evaluated the change in removal efficiencies of total organic carbon (TOC) and ammonia, microbial biomass, alkaline phosphatase (AKP) activity, microbial community structure, and AKP-associated genes. TOC and ammonia removal efficiency was highest in IP (64.8%) and cNMP (52.3%) treatments. Microbial community structure changed significantly across phosphorus sources that IP and cNMP encouraged *Enterobacter* and *Aeromonas*, respectively. The abundance of *phoA* and *phoU* genes was higher in IP treatments, whereas *phoD* and *phoX* genes dominated OP treatments. Our findings suggested that the performance of WWTPs was dependent on phosphorus sources and provided new insights into effective WWTP management.

## Introduction

Eutrophication is a serious problem among developing and developed countries (Hernández-Crespo et al., 2017), causing the degradation of aquatic environments, destruction of aquatic ecosystems and restrictions on drinking water supplies (Boeykens et al., 2017; Yu et al., 2017). Phosphorus is viewed as the most critical factor for eutrophication in most lake ecosystems attracting numerous studies (Achieng et al., 2017; Yu et al., 2017; Jiang et al., 2018). Sewage is one of the major source of phosphorus release into natural waters, and biological processes in wastewater treatment plants (WWTPs) contribute to phosphorus removal (Qiu et al., 2010; Zhao et al., 2016). More importantly, phosphorus sources are key in the stability and functions of the activated sludge, which is prone to swell owing to the non-filamentous bacteria in the absence of suitable phosphorus source in influents, consequently causing the declined MLSS and the poorer ability of removing ammonia and nitrite (Jobbágy et al., 2002). Among all phosphorus sources in the influent of WWTPs, inorganic phosphorus is of the most importance and Zhang et al. (2006) documented that the growth of sludge flocculent was declined when inorganic phosphorus was depleted, consequently causing the inhibited removal efficiencies of chemical oxygen demand (COD) and nitrogen. Although inorganic phosphorus was predominant in the activated sludge system, accounting for 49–87% of all the phosphorus sources from a study on 13 WWTPs in China (Peng et al., 2010), other phosphorus sources also played key roles in the stability and functions of the activated sludge. For instance, the settleability of the activated sludge system was significantly correlated with the concentration of adenosine triphosphate (ATP) (Clarke and Forster, 2010). Basically, the enhanced biological phosphorus removal (EBPR) mainly depends on the presence of phosphorus-accumulating organisms (PAOs), which uptake the excess available phosphorus under aerobic conditions, and trigger poly-phosphorus (poly-P) decomposition and poly-β-hydroxybutyrate (PHB) synthesis under anaerobic conditions (Feng et al., 2011; Jin et al., 2017). Additionally, PAOs remove ammonia and total organic carbon (TOC) simultaneously (Figdore et al., 2018). Nevertheless, most previous studies mainly focused on the removal of total phosphorus or orthophosphate by PAOs (Bassin et al., 2012; Zhao et al., 2016;Yang et al., 2017) or the simultaneous removal efficiencies of nitrogen and TOC with orthophosphate (Figdore et al., 2018), neglecting the impacts of phosphorus speciation in influents on wastewater treatment and the activated sludge. Since the activated sludge is composed of many microorganisms with their own phosphorus niche, microbial utilization of different phosphorus sources varies greatly (Bostrom et al., 1988), and potentially leads to the change of microbial diversity and functions. The impacts of phosphorus sources on the microbial structure and functions of the activated sludge in WWTPs are beyond our knowledge.

Of all the phosphorus sources, dissolved inorganic phosphorus is main one directly available for planktonic algae and bacteria (Bostrom et al., 1988). Organophosphate needs be converted to dissolved inorganic phosphorus before eventual utilization by planktons (Duhamel et al., 2010). To date, the three primary pathways of microbial organophosphate utilization include nucleotidase, carbon-phosphorus (C-P) lyase, and phosphatase (Zimmermann, 1992; Wanner, 1994; Dyhrman et al., 2006). The nucleotidase pathway has a strict substrate specificity, e.g., hydrolyzing nucleotides to produce phosphate and ribose or deoxyribose (Ammerman and Azam, 1991; Krug et al., 2016). C-P lyase can only metabolize phosphonates by rupturing the C-P bond (Ternan et al., 1998). Phosphatases are widely found in many organisms and viewed as the most common pathway for the whole ecosystems to obtain orthophosphate from organophosphates (OP) (Rodrigues et al., 2006). Phosphatase catalyzes the liberation of orthophosphate and organic residues from OP (Jansson et al., 1988), or induces the dephosphorylation by hydrolyzing the phosphate monoester bond (C-O-P) (Fisher, 1992). Phosphatases are extracellular enzymes categorized into three types depending on the substrate specificity, including phosphomonoesterase, phosphodiesterase and phosphotriesterase (Tiwari et al., 2015). Alternatively, they can be also classified as alkaline phosphatase (AKP) and acid phosphatase (ACP) based on the optimum pH (Xie et al., 2010). ACP is found within several strains (Li et al., 2017) but only a few studies have reported the variation of ACP activities responsive to different phosphorus concentration (Kruskopf and Plessis, 2004). AKP is characterized as a typically inducible enzyme and generally considered to be non-specific and extracellular (Song et al., 2018). Numerous studies have recognized that AKP activities in aquatic system are related to eutrophication (Dai et al., 2018). Accordingly, AKP is the most extensively investigated phosphatase, and its encoding genes and target substrates are well studied (Song et al., 2007; Lin et al., 2015). Phosphate-specific transporter (*Pst*) system is critical in adapting to phosphorus niche, encoding different enzymes with high affinity to recognize and catalyze various OP under orthophosphate-depleted conditions (Ternan et al., 1998). Among them, PhoA is a phosphomonoesterase targeting phosphate monoesters mainly identified in *Bacteroidetes* and *Chloroflexi*. Another two common AKP-encoding genes are *phoD* (*Aphanothece halophytica* and *Anabaena* sp.) and *phoX* (*Proteobacteria*, *Actinobacteria*, *Bacteroidetes*, and *Cyanobacteria*) (Dai et al., 2016). Either PhoD or PhoX can target phosphate monoesters and phosphodiesters, behaving as both phosphomonoesterase, and phosphodiesterase (Dai et al., 2016). Additionally, *phoU* and *phoR* genes encode the repressors for *Pst* system regulated by inorganic phosphate (Ternan et al., 1998).

Till now, the occurrence and functions of phosphatase across organisms targeting a narrow spectrum of OP have been well studied, but there are few reports focusing on the impacts of different phosphorus sources on the microbial community structure or AKP-encoding gene diversity in the activated sludge from WWTPs. The relationship remains unclear between phosphorus source and microbial growth, AKP activity, or contaminant removal efficiency in WWTPs. This work aimed to fill in these gaps by analyzing the change in removal efficiencies of TOC and ammonia, microbial growth, AKP activities, relative abundance of AKP-associated genes, and microbial community structure in an activated sludge cultivated with 59 different phosphorus sources in a high-throughput manner. Our findings will unravel the comprehensive link of phosphorus niche to microbial structure and functions in the activated sludge, providing deeper understanding on influential factors in wastewater treatment and offering theoretical supports for WWTPs management.

## Materials and Methods

### Experimental Design and Operation

The activated sludge used in this study was inoculated in July 2017 from an operating activated sludge reactor located in Beijing Normal University, Beijing, China. They were further cultivated in a sequencing batch reactor (SBR, 10 L) for 90 days at room temperature with 7 days of sludge retention time, 18 h of hydraulic retention time, 12 h of one cycle and pH between 6 and 8. The influent water quality was as follows: TOC of 275.9 ± 1.73 mg/L, ammonia (NH_3_-N) of 48.96 ± 1.29 mg/L, total nitrogen (TN) of 51.47 ± 0.82 mg/L, and total phosphorus of 4.97 ± 0.02 mg/L. A stable effluent quality was achieved (Supplementary Table S1) when the mixed liquor suspended solids (MLSS) of the activated sludge reached 3.5 ± 0.1 g/L.

Phosphorus-free M9 medium were used to cultivate the activated sludge (Supplementary Material), adjusted to pH approximately 7.0, and autoclaved (Akhtar and Rehman, 2017). The Phenotype Microarray 4A (PM4A Microplate, Biolog Inc., United States) was used to investigate the impacts of phosphorus sources on the activated sludge in a high-throughput manner, which contained 59 different phosphorus sources in each well. Based on the chemical structures, they were categorized into four groups as: 7 inorganic phosphorus (IPs), 14 nucleoside monophosphates (NMPs), 9 cyclic nucleoside monophosphates (cNMPs), and 29 other OPs, details in Supplementary Figure S1. After adding 100 μL of sterile water into each well of the PM4A Microplate and mixed well, 30 μL of each solution was transferred into a 96-well cell-culture microplate, supplemented with 195 μL of phosphorus-free M9 medium, and 25 μL of the activated sludge (final MLSS = 0.076 g/L). The cell-culture microplate was then inoculated in an incubator (HPS-250, Donglian Electronic Technology Development Company of Harbin, China) at 30°C for 3 days. All the cultivation was carried out in triplicates. Water samples were collected at 0, 3, 6, 12, 24, 36, 48, 60, and 72 h for the measurement of optical density at 620 nm (OD_620_). At 0 and 72 h, extra water samples were collected for the measurement of orthophosphate concentration and AKP activities. The three replicates were combined for DNA extraction to analyze the microbial community structure and relative abundance of AKP-associated genes.

### Chemical Analysis

For the influents and effluents of SBR, the measurement of MLSS, NH_3_-N, TN, and total phosphorus followed standard methods (American Public Health Association [APHA], 1998). TOC was measured by a TOC analyzer (vario TOC cube, Elementar, Germany). Orthophosphate concentration was determined by the modified molybdenum blue spectrophotometric method (Vieira and Nogueira, 2004). Details for MLSS, TOC, ammonia and orthophosphate measurement are described in Supplementary Material.

### Biological Analysis

Alkaline phosphatase activities were measured using the EnzChek^®^ Phosphatase Assay Kit (Molecular Probes, United States) following manufacturer’s instruction (Zhu et al., 2016; details in Supplementary Material). DNA was extracted from the activated sludge using the TIANamp Bacteria DNA Kit (Tiangen Biotech, China) according to manufacturer’s protocols (Details in Supplementary Material). The V3-V4 hypervariable region of bacterial 16S rRNA genes were amplified by polymerase chain reaction (PCR) for sequencing with the primer set of 338F (5-ACTCCTACGGGAGGCAGCAG-3) and 806R (5-GGACTACHVGGGTWTCTAAT-3) (Munyaka et al., 2015; details in Supplementary Material). Sequences were assembled based on overlap and assigned to samples according to their unique barcode tag. Then, singletons were discarded to reduce the error rate with a small reduction in sensitivity. The representative sequence set was then selected and aligned, and chimeric sequences identified by the UCHIME algorithm were discarded (Edgar et al., 2011). Operational taxonomic units (OTUs) with 97% similarity were selected using UCLUST. The quantitative insights into microbial ecology (QIIME) was used to classify all sequences into different taxonomic groups (Cole et al., 2009; Luria et al., 2014). The relative abundance of each taxon and OTU within each community was calculated by comparing its number of sequences with the number of total sequences. The indices of α-diversity (Shannon and Chao1) were calculated using Mothur to analyze the complexity of species diversity, after randomly selecting 20,000 reads from each sample.

Four AKP-associated genes were investigated, including three AKP-encoding genes (*phoA*, *phoD*, and *phoX*) (Ternan et al., 1998; Apel et al., 2007; Dai et al., 2014, 2015, 2016; Ragot et al., 2015; Fraser et al., 2017) and one AKP-repressor-encoding gene (*phoU*) (Sang et al., 2014). Their abundance was measured by quantitative PCR with degenerate primers according to previous studies (Supplementary Table S2; Luo et al., 2009; Sebastian and Ammerman, 2009; Sang et al., 2014; Fraser et al., 2015) on the ABI7500 (Applied Biosystems, United States). The reaction mixture contained 1 μL of DNA template, 1 μL of each primer, 25 μL of PremiSTAR HS and 22 μL of H_2_O. The PCR was performed under the following conditions: 95°C for 5 min; 30 cycles of 94°C for 30 s, 55°C for 30 s and 72°C for 30 s; a final extension at 72°C for 10 min. The recombinant sequences containing the four AKP-associated genes was extracted from pGEM-T vector in *E. coli* and serially diluted (from 10^8^ to 10 copies) to obtain standard curves.

### Data Analysis

Alkaline phosphatase activity (U/L) was calculated according to the calibration curve (*R*^2^ = 0.9996) with the following Equation (1).

AKP =(A.U.−6.233)/6432.8

where *A.U.* is the fluorescence intensity (460 nm) measured by the microplate reader (Synergy 2, Gene Company Ltd., United States).

All the data are mean ± standard deviation (SD). Statistical analysis was performed using SPSS software (version 20.0). Redundancy analysis (RDA) was processed and plotted by Canoco 4.5 for the correlations between bacterial community structure, diversity, AKP-encoding genes, phosphorus sources, AKP activities, MLSS, residual orthophosphate, and removal of TOC and ammonia.

## Results and Discussion

### Removal of TOC and Ammonia Across Phosphorus Sources

The removal efficiencies of TOC and ammonia varied significantly across 59 phosphorus sources after 72 h cultivation. Without phosphorus source (well A1), TOC and ammonia removal efficiency was the lowest (40.2 ± 0.1% and 33.7 ± 0.2%, respectively). Generally, TOC removal efficiency ranged from 40.9 ± 0.1% to 78.0 ± 0.1% across phosphorus sources, and the activated sludge cultivated with orthophosphate (78.0 ± 0.1%) and tripolyphosphate (77.4 ± 0.2%) had the highest TOC removal efficiency. Additionally, TOC removal efficiency was lower in D-mannose-1-phosphate (40.9 ± 0.1%), inositol hexaphosphate (47.5 ± 0.1%), and hypophosphite (48.0 ± 0.2%). In summary, IP achieved the highest average TOC removal efficiency (64.8 ± 13.1%), followed by cNMP (60.5 ± 6.9%), NMP (59.0 ± 5.6%) and OP (57.0 ± 7.5%), but showing no significant difference (*p* > 0.05).

Ammonia removal efficiency of the activated sludge ranged from 34.1 ± 0.1% to 85.8 ± 0.1% across phosphorus sources. The highest ammonia removal efficiency was achieved in the treatments of thymidine-3′,5′-cyclic-monophosphate (85.8 ± 0.1%) and uridine-3′-monophosphate (85.6 ± 0.2%). The activated sludge cultivated with D-glucose-6-phosphate (30.8 ± 0.2%), thiophosphate (34.1 ± 0.1%), tripolyphosphate (34.4 ± 0.1%), adenosine-2′-monophosphate (34.8 ± 0.1%), hypophosphite (35.2 ± 0.2%), guanosine-3′-monophosphate (36.5 ± 0.1%), adenosine-3′-monophosphate (38.6 ± 0.2%), D-mannose-1-phosphate (38.9 ± 0.3%), and adenosine-5′ monophosphate (39.1 ± 0.1%) had the lowest ammonia removal efficiency. The activated sludge cultivated with cNMP and OP had significantly higher ammonia removal efficiencies (*p* < 0.05), averagely 52.3% ± 6.4 and 51.2 ± 6.6%, than NMP (47.6 ± 5.1%), and IP (35.5 ± 4.8%).

### Growth of the Activated Sludge Cultivated With Different Phosphorus Sources

**FIGURE 1:**
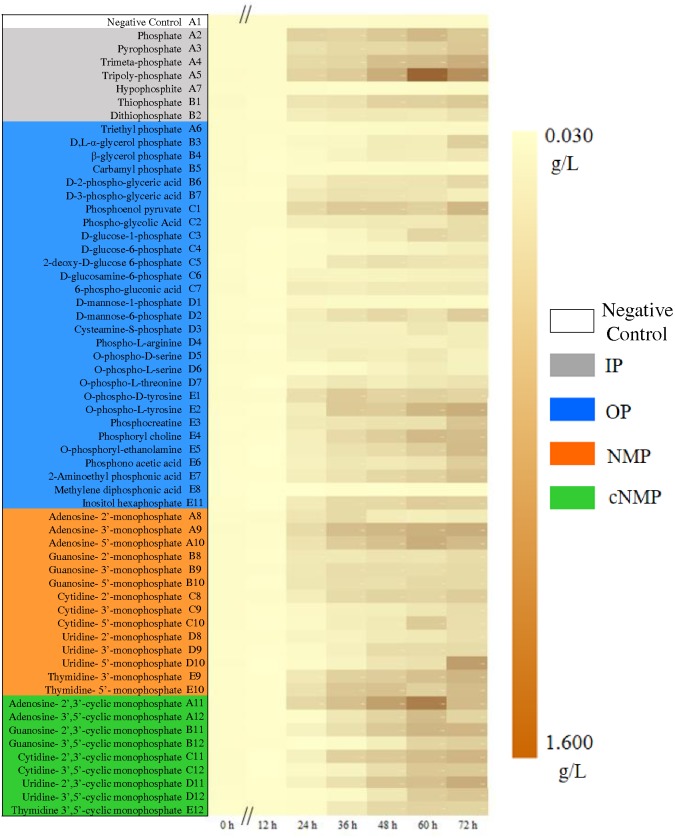
Growth dynamics of the activated sludge cultivated with different phosphorus sources. Phosphorus sources with different background color refer to different groups: Gray for IP, blue for OP, brown for NMP, green for cNMP, and white for negative control. MLSS increases from 0.030 g/L (yellow) to 1.600 g/L (brown).

After 72-h cultivation, the growth curves of the activated sludge exhibited significant difference (Figure 1). The MLSS remained extremely low without phosphorus source throughout the cultivation period (well A1, 0.067 ± 0.019 g/L). In the first 24 h, an obvious lag phase was observed for all the treatments. The exponential phase started from 24 h, expect for carbamyl phosphate (well B5) and methylene diphosphonic acid (well E8). The best growth of activated sludge was supported by cNMP, and the MLSS achieved averagely 0.617 g/L. All the MLSS in NMP or cNMP treatment was higher than 0.229 g/L, and the highest MLSS was in uridine-5′-monophosphate (0.982 g/L, NMP), and uridine-2′,3′-cyclic monophosphate (0.846 g/L, cNMP). The activated sludge cultivated with IP had moderate MLSS (0.574 g/L), e.g., orthophosphate (0.562 g/L) and tripolyphosphate (1.119 g/L). The MLSS in NMP (0.517 g/L) and OP (0.357 g/L) treatments were relatively lower.

These results suggested that most IPs in the present study supported the microbial growth of the activated sludge, explained by the well-known fact that aquatic microorganisms can directly utilize dissolved inorganic phosphorus (Reynolds, 1997). Unexpectedly, both NMPs and cNMPs benefited higher microbial growth than IPs. It was possibly attributed to the organic moiety released by NMPs and cNMPs after dephosphorylation, evidenced by the dependent growth efficiency of planktonic microorganisms on the chemical form of available carbon (Schweitzer and Simon, 1995). Accordingly, dephosphorylating NMPs and cNMPs provided both inorganic phosphorus and extra carbon sources, contributing to better microbial growth than IP. Similar findings were observed for *Prochlorococcus marinus* PCC 9511, whose growth rate was higher when cultivated with OP than orthophosphate, including β-glycerol-phosphate, pyrophosphate, glucose 6-phosphate, and ATP (Rippka et al., 2000). Higher growth rates of *Cylindrospermopsis raciborskii* were obtained when cultivated with D-glucose-6-phosphate and β-glycerol-phosphate than orthophosphate (Bai et al., 2014).

Some phosphorus sources could not be effectively used by the activated sludge and the MLSS maintained below 0.1 g/L. Among them, hypophosphite treatment only had the MLSS of 0.080 g/L, possibly attributing to its strong reducibility (Gan et al., 2007). Others were OPs, including carbamyl phosphate, methylene diphosphonic, triethyl phosphate and D-mannose-1-phosphate. Carbamyl phosphate was readily decomposed into cyanate and orthophosphate, which might lead to toxic cyanate accumulation (Leibold and McPeek, 2006). Methylene diphosphonic has the C-P bond and can be only metabolized *via* the C-P lyase pathway, not as abundant as AKP (Ternan et al., 1998; Borisova et al., 2011). Triethyl phosphate does not support microbial growth and even behaves toxic to some microorganisms (Wang et al., 2011). D-mannose-1-phosphate is seldom reported to be utilized by microbes and the only study by Martins confirmed that it can support faster growth of some organisms under acidic conditions (Martins et al., 2013). Our results were consistent with these previous studies and suggested that different phosphorus sources have diverse utilization profiles by microbes in the activated sludge.

### AKP Activities and Residual Orthophosphate in the Activated Sludge

**FIGURE 2:**
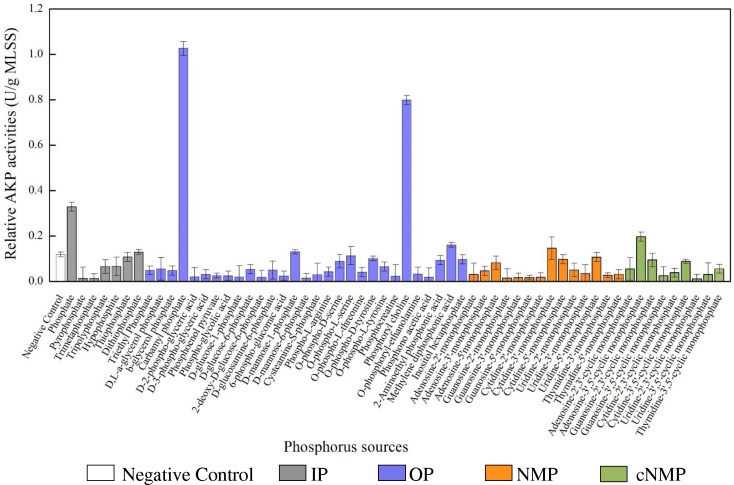
The specific AKP activities cultivated with different phosphorus sources (white bar for negative control, gray bar for IP, blue bar for OP, brown bar for NMP, and green bar for cNMP). Data are mean ± standard deviation.

Similar with the MLSS, the specific AKP activities also varied across phosphorus sources (Figure 2). Compared to the control, 80% of the phosphorus sources had higher specific AKP activities. Among them, the specific AKP activities were higher in IP treatments (0.090 U/g MLSS) and OP groups (0.091 U/g MLSS), followed by cNMP (0.065 U/g MLSS) and NMP (0.031 U/g MLSS). To be more precise, orthophosphate-cultivated sludge had higher specific AKP activity (0.329 U/g MLSS), and other phosphorus sources with higher specific AKP activity were carbamyl phosphate (1.026 U/g MLSS) and phosphoryl choline (0.798 U/g MLSS). Some previous studies have reported similar patterns of the AKP activities of pure strains across different phosphorus sources. For instance, *Bacillus cereus* had higher AKP activities at low phosphate concentration than under phosphorus-free conditions (Vinter et al., 1987). The AKP activity of cyanobacterial *Nodularia spumigena* was highest in the absence of orthophosphate and was only upregulated postexposure to 2-aimonmethylphosphonate rather than methylphosphonate or ethylphosphonate (Teikari et al., 2018). In contrast, the lowest specific AKP activities were observed in the treatments of 2-deoxy-D-glucose-6-phosphate (0.016 U/g MLSS) and triethyl phosphate (0.047 U/g MLSS). The strong toxicity of triethyl phosphate (Wang et al., 2011) might explain the poor specific AKP activities.

After 72-h cultivation, the residual orthophosphate in the negative control (0.128 mg/L) was much lower than that in orthophosphate treatment (well A2, 0.897 mg/L), suggesting neglectable orthophosphate came from the activated sludge and most of the residual orthophosphate was released from phosphorus sources *via* microbial utilization (Supplementary Figure S2). The highest residual orthophosphate concentration was achieved in tripolyphosphate treatment (7.564 mg/L), and the lowest ones (averagely 0.128 mg/L) were among the activated sludge with low MLSS, including hypophosphite, dithiophosphate, triethyl phosphate, and D-mannose-1-phosphate.

### Bacterial Community Structure of the Activated Sludge Cultivated With Different Phosphorus Sources

A total of 1,776,562 quality-filtered, chimera-free sequences were obtained, ranging from 17,937 to 39,967 in different treatments. They were assigned to 201 distinct OTUs at 97% identity. IP treatments had the highest OTU numbers (155), followed by OP (131), NMP (129), and cNMP (103), as listed in Supplementary Table S3.

**FIGURE 3:**
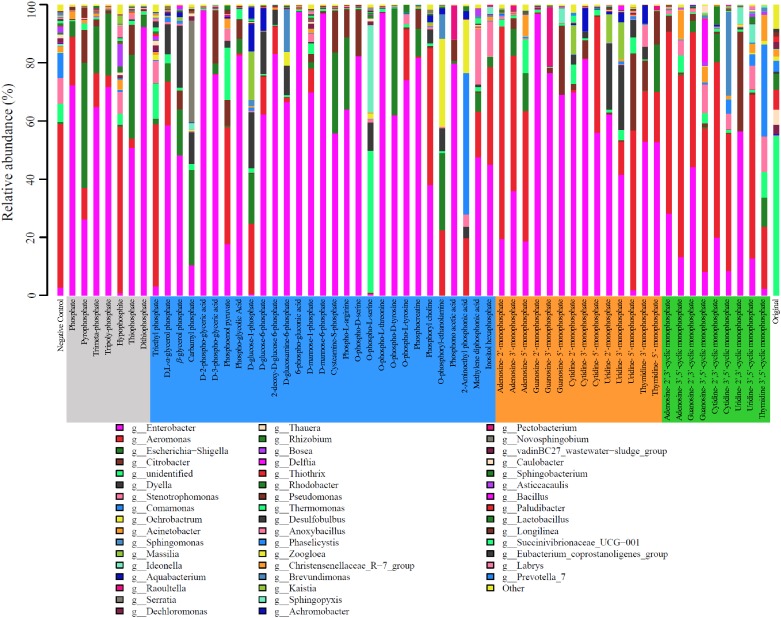
Relative abundance of microbes on genus level in the activated sludge cultivated with different phosphorus sources.

The microbial community of the activated sludge was consisted of the classes *Alphaproteobacteria*, *Betaproteobacteria*, *Deltaproteobacteria*, *Gammaproteobacteria*, and *Bacilli*. In the original activated sludge, *Sphingobacteriia* (22.52%), *Betaproteobacteria* (15.87%), and *Bacteroidia* (12.84%) were dominant bacterial classes, whereas *Gammaproteobacteria* dominated the sludge after cultivation with all types of phosphorus sources, reaching 93.90, 88.70, 91.47, and 82.96%, respectively. At genus level, 201 bacterial lineages were observed and the most abundant 49 genera (>1%) are illustrated in Figure 3. In the original activated sludge, the dominant bacterial lineages were *Thiothrix* (18.65%), *Rhodobacter* (14.64%), and *Thauera* (14.01%). *Enterobacter* became the dominant genus IP, OP and NMP treatments (averagely 58.36, 58.49, and 54.93%, respectively). Particularly for D-2-phospho-glyceric acid, guanosine-2′-monophosphate, 6-phospho-glucomic acid and O-phospho-L-threonine, the relative abundance of *Enterobacter* was even above 98%. *Aeromonas* was dominant in cNMP treatments (averagely 45.32%), especially for adenosine-3′,5′-cyclic monophosphate (67.33%), adenosine-2′,3′-cyclic monophosphate (63.00%), and cytidine-2′,3′-cyclic monophosphate (61.23%). The dominance of *Enterobacter* and *Aeromonas* was attributed to their important roles in phosphorus utilization as denitrifying phosphorus-accumulating bacteria (DPAB) (Wan et al., 2017).

**FIGURE 4:**
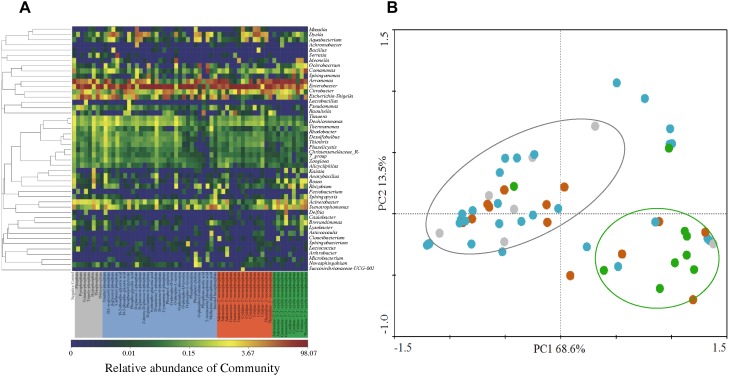
**(A)** Heatmap of microbial community structures of the activated sludge cultivated with different phosphorus sources. **(B)** PCA score plot of the bacterial lineages on genera level in the activated sludge cultivated with different phosphorus sources. Gray, blue, brown, and green dots represent the microbial community of the activated sludge cultivated with IP, OP, NMP, and cNMP, respectively.

Two bacterial genera with relatively higher abundance across all the treatments included *Citrobacter* (3.33–5.98%) and *Escherichia-Shigella* (2.50–16.23%). Figure 4A illustrates the heatmap of microbial community structures of the activated sludge. The relative abundance of *Dyella* was higher in NMP treatments and *Comamonas* had higher abundance in cNMP treatments. For the OP treatments, *Ochrobactrum* and *Serratia* were the unique dominant bacteria. However, no previous study has reported their roles in phosphorus cycling and the reason for their occurrence in the present study is unclear. Principal components analysis (PCA) score plot (Figure 4B) further confirmed our findings that the microbial communities of IP (gray dots), OP (blue dots) and cNMP (green dots) treatments were clustered together and separated from each other, illustrating significant differences in community composition. In contrast, the microbial communities of NMP (brown dots) treatments had more diverse distribution. These results indicated the distinct impacts of IPs, OPs, and cNMPs on microbial community structure of the activated sludge.

**FIGURE 5:**
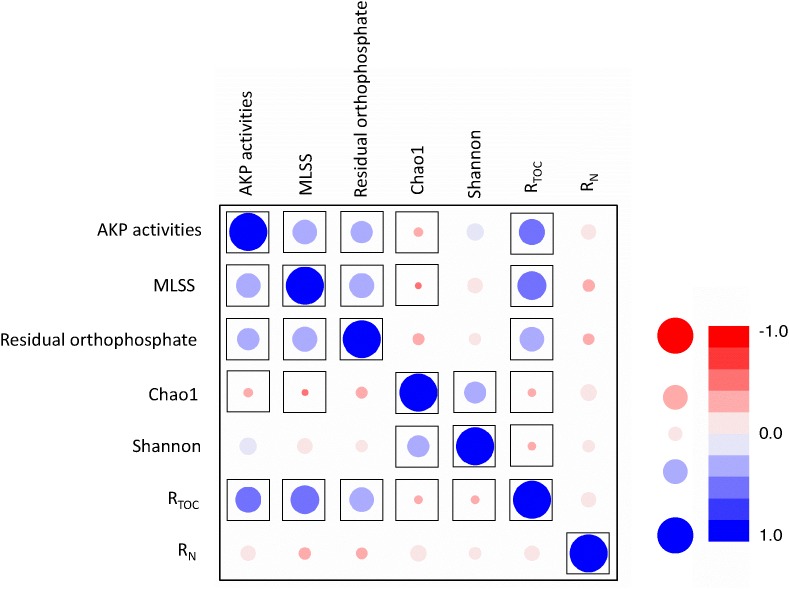
Correlation analyses between MLSS, AKP activity, residual orthophosphate, TOC removal, and ammonia removal. R_N_ represents ammonia removal efficiency and R_TOC_ represents TOC removal efficiency. The color shading and bubble size refer to linear coefficients, and framed points represent the significant correlation with *p* < 0.05.

Two indices of α-diversity, Shannon and Chao1, were used to analyze the complexity of species diversity across different phosphorus sources (Supplementary Table S3). The negative control had significantly higher Chao 1 (313.08 ± 18.36) and Shannon (3.17 ± 0.02) indices than other treatments, indicating sole phosphorus source addition reduced the diversity of microbial community in the activated sludge. This result was consistent with Beauregard et al. (2010) work that the long-term phosphorus fertilization decreased the microbial α-diversity in the rhizosphere. The significant decline of α-diversity was possibly explained by the selective pressure induced by the competitive advantage of the microbes capable of utilizing the sole phosphorus (Kong et al., 2017). IP treatments had the highest species richness (Chao 1 = 192.75 ± 55.62), followed by NMP (Chao 1 = 178.92 ± 40.48), and OP (Chao 1 = 164.80 ± 73.27). It is worth mentioning that Chao 1 was higher in triethyl phosphate, D-mannose-1-phosphate and O-phospho-L-serine of OP, reaching 350.29 ± 25.14, 326.83 ± 18.31, and 297.84 ± 26.97, respectively. In contrast, Shannon indices of cNMP treatments were higher (2.13) than those of IP treatments (1.82). We also observed a negative correlation between the MLSS and Chao 1 index (Figure 5, *r* = -0.463, *p* < 0.01), consistent with a previous report that the species richness decreased with the growth of the activated sludge (Kong et al., 2017). It suggested some genera preferentially utilizing the supplied phosphorus sources might dominate the microbial community and declined the species diversity of the activated sludge.

### Abundance of AKP-Associated Genes in the Activated Sludge Cultivated With Different Phosphorus Sources

The abundance of AKP-associated genes, including *phoA*, *phoX*, *phoD* and *phoU*, is illustrated in Supplementary Figure S3. *phoA* gene had the highest abundance (3.04 ± 0.36 × 10^5^ to 51.9 ± 0.5 × 10^5^ copies/g MLSS), followed by *phoD* (0.05 ± 0.01 × 10^5^ to 16.3 ± 2.0 × 10^5^ copies/g MLSS) and *phoX* (2.66 ± 0.25 × 10^3^ to 50.9 ± 0.1 × 10^3^ copies/g MLSS). The abundance of *phoU* gene encoding *Pst*-repressor ranged from 6.57 ± 0.04 × 10^4^ to 41.9 ± 0.8 × 10^4^ copies/g MLSS.

Across different phosphorus sources, the abundance of *phoA* and *phoU* genes had a positive linear correlation (*p* < 0.01, Supplementary Figure S4), both higher in the activated sludge cultivated with IPs and some OPs (2-deoxy-D-glucose-6-phosphate and O-phosphoryl-ethanolamine). It might be attributed to their co-occurrence, evidenced by previous findings that *phoA* and *phoU* genes are always clustered in operons, e.g., *pstSCAB-phoU* (Steed and Wanner, 1993) and *pho* (Vershinina and Znamenskaya, 2002). *Pst* operon plays an important role when orthophosphate is limited (Torriani, 1990), and *phoU* gene is found in a wide range of bacterial genera, including *Escherichia*, *Pseudomonas*, and *Acinetobacter* (Hirota et al., 2013). Our results hinted the key roles of *phoA* gene encoding AKP in the activated sludge in utilizing phosphorus sources in IP and OP treatments, suggesting that the *phoA*-harboring bacteria might obtain more competitive advantages in phosphorus-depleted environments and alter the microbial communities.

The abundance of *phoD* gene was similar across all the treatments with no remarkable difference (*p* > 0.05). It is worth mentioning that *phoX* gene varied significantly. Its abundance was about one order of magnitude higher (averagely 2.08 × 10^4^ copies/g MLSS) in the treatments of cytidine monophosphate, uridine monophosphate, β-glycerol phosphate, phospho-L-arginine, O-phospho-D-tyrosine, and O-phosphoryl-ethanolamine than other phosphorus source treatments (averagely 5.12 × 10^3^ copies/g MLSS). *phoD* and *phoX* genes are found in different bacterial lineages. Dominant *phoD*-harboring phyla included *Proteobacteria* and *Firmicutes*, whereas *phoX* genes were found in *Proteobacteria* (Ragot et al., 2017; Hu et al., 2018). The *phoX*- and *phoD*-encoding AKP is reported to exhibit promiscuous enzymatic activities on OPs (Lin et al., 2015). PhoD has lower substrate specificity to organophosphate, and thus *phoD*-harboring bacteria could utilize a huge range of OP. Similarly, PhoX behaved lower substrate specificity for C-O-P bonds (Zaheer et al., 2009). Of all the 59 phosphorus sources, cytidine monophosphate, uridine monophosphate and some OPs mentioned above might have the unique branched-chain structure associated with C-O-P. Therefore, *phoD*- or *phoX*-harboring bacteria could possibly thrive in the treatments with these specific phosphorus sources, offering an explanation on the distribution of AKP encoding gene phylotypes.

### Correlations Between TOC Removal, Ammonia Removal, Residual Orthophosphate, MLSS, and AKP Activity

Pearson’s correlation analysis unraveled the relationships between TOC removal, ammonia removal, MLSS, AKP activity, and residual orthophosphate (Figure 5 and Supplementary Table S4). The MLSS and residual orthophosphate were positively correlated (*p* < 0.01). As orthophosphate could be directly utilized by microbes, our results were consistent with some previous findings about the positive correlation between orthophosphate concentration and phytoplanktonic yield under phosphorus-depleted conditions (Ryther and Dunstan, 1971).

There was also a positive correlation between the AKP activity and MLSS (*p* < 0.01, Figure 5). As extracellular enzymes in many microorganisms (Rodrigues et al., 2006), higher AKP activities to liberate orthophosphate led to more effective utilization of phosphorus sources and higher MLSS. Similar results were reported that AKP activities were closely related to bacterial biomass (Vinter et al., 1987), but not orthophosphate (Labry et al., 2016).

It is worth mentioning a positive relationship between the residual orthophosphate concentration and AKP activity (*p* < 0.05, Figure 5), inconsistent with previous studies. The well-accepted theory is the negative regulation of the AKP-encoding gene expression and AKP activities by orthophosphate (Dyhrman and Palenik, 1999; Beardall et al., 2001; Dyhrman and Ruttenberg, 2006). The AKP activities of *Bacillus cereus* were repressed by orthophosphate (Vinter et al., 1987). Orthophosphate depletion could induce AKP activities in *Emiliania huxleyi*, but the impacts of D-L-α-glycerophosphate and adenosine monophosphate (AMP) were not mentioned (Dyhrman and Palenik, 2003). Nevertheless, some other studies reported contradictory results. In Xie et al. (2010) work, the AKP activity of the activated sludge was intensively inhibited by pyrophosphate, whereas the inhibition by orthophosphate was limited. Several reasons might explain the different relationships between orthophosphate concentration and AKP activities. Firstly, most studies on the effects of orthophosphate on AKP activity addressed individual strains (Rodrigues et al., 2006; Fu et al., 2013), and the mechanisms of orthophosphate regulating AKP-encoding genes might vary across bacterial species. Secondly, only limited IPs or OPs have been studied, e.g., orthophosphate and ATP (Xie et al., 2010); there is lack of a precise database for broader spectra of phosphorus sources in a high-throughput manner. The regulation of AKP-encoding gene expression is possibly dependent on phosphorus sources (Vinter et al., 1987; Xie et al., 2010). Finally, the release of orthophosphate by hydrolysing phosphorus sources and the suppression of AKP activities induced by orthophosphate is a balance of feedback regulation (ChrȮst and Overbeck, 1987;Ghyoot et al., 2015). In this study, most of the orthophosphate came from the hydrolysis of phosphorus sources and this process was more predominant than the suppression effect induced by orthophosphate. Hence, their relationship behaved positively.

Here, we used PM4A Microplate as a high-throughput method to investigate the impacts of 59 phosphorus sources on the AKP activities of the activated sludge and found they were affected by many environmental variables. For instance, although AKP activity was repressed in the presence of orthophosphate (Thompson and Chassy, 1982), it was more correlated with microbial biomass (Vinter et al., 1987). In the present study, the activated sludge with the highest AKP activity (orthophosphate, 0.165 U/L; phosphoryl choline, 0.146 U/L; tripolyphosphate, 0.107 U/L) also had higher MLSS. Alternatively, some OP can inhibit cell growth and eventually decrease AKP activities, e.g., 2-deoxy-D-glucose-6-phosphate (Thompson and Chassy, 1982) and hypophosphite (Gan et al., 2007). Similar results were found in this study that the activated sludge cultivated with hypophosphite, 2-deoxy-D-glucose-6-phosphate, and triethyl phosphate had both lowest AKP activities and MLSS.

TOC removal efficiency and MLSS were positively correlated (*p* < 0.01), explained by the fact that TOC removal is a process of transformation from TOC to biomass and energy, evidenced by some previous studies (Wang et al., 2012). TOC removal also had positive correlations with AKP activities and residual orthophosphate (*p* < 0.01), but was negatively correlated with Chao 1 and Shannon indices (*p* < 0.01). This result suggested the dominance of specific microbes capable of utilizing sole phosphorus source which might replace the ecological niche of other microbes in the activated sludge. Our results revealed a crucial role of phosphorus availability in TOC removal. Unexpectedly, the ammonia removal behaved no direct relationship with phosphorus sources.

**FIGURE 6:**
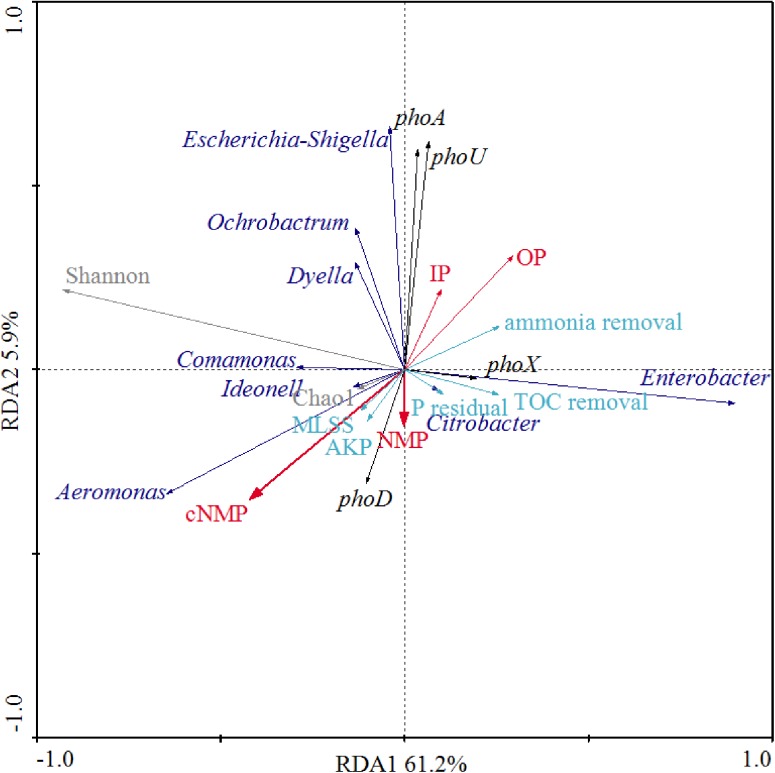
Redundancy analysis (RDA) between phosphorus sources, MLSS, AKP activity, residual orthophosphate, microbial diversity indices, dominant bacterial lineages, AKP-associated genes, TOC removal, and ammonia removal. Red arrows present different phosphorus sources; cyan arrows represent MLSS, AKP activity, residual orthophosphate, TOC removal, and ammonia removal; black arrows represent the abundance of AKP-associated genes; gray arrows represent microbial diversity indices (Chao1 and Shannon); blue arrows represent dominant bacterial lineages.

We further examined the correlations between phosphorus sources and the other physiological parameters and dominant bacterial lineages of the activated sludge, as illustrated in Figure 6. Since detrended correspondence analysis (DCA) showed that the lengths of first ordination gradient were less than 3, RDA was adopted in this study. The eigenvalues of the first two axes were 0.612 and 0.058, respectively, which explained 67.1% of the total variance. There were positive relationships between NMPs, cNMPs, MLSS and AKP activities, consistent with our findings above about the linear correlation between MLSS and AKP activities (Figure 5) and higher AKP activities in cNMP treatments (Figure 2). Some previous studies found higher growth rate of *P. marinus* PCC 9511 when cultivated with OP than orthophosphate (Rippka et al., 2000) and the growth-dependent AKP activities (Vinter et al., 1987; Labry et al., 2016). TOC removal was positively related with residual orthophosphate, consistent with Figure 5. It is worth noting that the AKP activities were negatively correlated with IPs and OPs.

As *Aeromonas* was the dominant bacterial lineage in cNMP treatments, its abundance was positively correlated with cNMPs. There is no previous study investigating the utilization of cNMPs by *Aeromonas*. The abundance of *phoA* gene was positively correlated with *Escherichia-Shigella*, hinting that it might be *phoA*-harboring bacterial lineage (Vershinina and Znamenskaya, 2002). The positive correlation between the abundance of *phoX* gene and *Enterobacter* also suggested it as a *phoX*-harboring bacterial lineage. *phoX* gene was previously found in *Proteobacteria* (Ragot et al., 2017), but there is no previous study asserting its occurrence in *Enterobacter*. The abundance of *phoD* gene was positively correlated with NMPs and cNMPs, strongly hinting that phosphorus sources could promote different bacteria with *phoX* or *phoD* genes, and *phoX*- and *phoD*-encoding AKPs were responsible for the dephosphorylation of NMPs or cNMPs. These results suggested that the impacts of phosphorus sources on microbial community in activated sludge were attributed to the distinct phosphorus utilization profiles of different microbes.

### Roles of Phosphorus Sources in Shaping Microbial Communities

Understanding the community assembly of the activated sludge is crucial for improving WWTPs performance and exploring the mechanisms of microbial ecology (Zhang et al., 2015; Xia et al., 2018). Community assembly is a process highly influenced by trophic conditions (Caruso et al., 2011) and the two well-accepted theories include neutral theory addressing stochastic process and niche theory highlighting importance of deterministic processes (Leibold and McPeek, 2006). In the present study, the significant impacts of phosphorus sources on microbial structure and functions strongly hinted that the deterministic process was more responsible in structuring microbial community in the activated sludge, and niche theory explains the roles of phosphorus sources better. The composition and diversity of microbial community and AKP-associated genes in the activated sludges were dependent on the phosphorus source. It is explained by the role of individual phosphorus source in selecting populations capable of utilizing the supplemented phosphorus source, evidenced by the distinct enrichment of specific microbes and AKP-associated genes (Figure 3 and Supplementary Figure S3). The selective power which altered the microbial community can be originated from niche filtering (Chase and Myers, 2011). Similar with a previous study on soybean rhizosphere that phosphorus availability induced the niche filtering process (Mendes et al., 2014), phosphorus sources markedly decreased the community complexity comparing to control. For instance, the microbial diversity in the activated sludge cultivated with different phosphorus source groups followed the order: IP > OP > NMP > cNMP, proving that the selective power was dependent on phosphorus sources. Additionally, IP can be utilized by most microbes, maintaining the highest microbial diversity, whereas cNMPs can only be readily used by specific bacterial lineages, consequently driving the lowest microbial diversity. As for NMP and OP, their utilization by microbes is more difficult than cNMPs and they thus hold moderate microbial diversity. Accordingly, we speculated that phosphorus sources with higher utilization specificity can drive stronger niche filtering process.

In the present study, the activated sludge was cultivated with 59 phosphorus sources and we evaluated several physiological indices and community structure to show their intra-relationship. Phosphorus sources had significant effects on the MLSS, AKP activity, microbial community structure, and abundance of AKP-associated genes in the activated sludge. Although IP supported better microbial growth, cNMPs were the most favorable phosphorus sources utilized by most of the microorganisms. In addition, microbes may have more ways to utilize NMP and cNMP than OP. The four groups of phosphorus sources (IP, OP, NMP, and cNMP) drove the niche-based selection, leading to the difference in the dominant bacterial lineages and the diversity of AKP-encoding genes across the activated sludge cultivated with different phosphorus sources. Our findings unravel the complicated impacts of phosphorus sources on the structure and functions of microbial community in a high-throughput manner, providing theoretical support for better understanding the phosphorus utilization in the activated sludge and improving performance and stability of WWTPs.

## Author Contributions

DZ and EX designed the study. LZ and MR conducted the experiments and interpreted the data. DZ, LZ, EX, YL, and SD contributed to data analysis. MR, EX, and DZ wrote the manuscript. SD, AD, and DZ revised the manuscript.

## Conflict of Interest Statement

The authors declare that the research was conducted in the absence of any commercial or financial relationships that could be construed as a potential conflict of interest.
