# Supplementary material for: Roles of Phosphorus Sources in Microbial Community Assembly for the Removal of Organic Matters and Ammonia in Activated Sludge
Source: Front Microbiol. 2019 May 16;10:1023. doi: 10.3389/fmicb.2019.01023 (PMC6532738; doi:10.3389/fmicb.2019.01023)
Supplement: Supplementary file 1 [file Data_Sheet_1.docx]

**Supporting Information**

**Roles of phosphorus sources in microbial community assembly for the removal of organic matters and ammonia in activated sludge**

Lei Zheng^a^, Mengli Ren^a^, En Xie^b,*^, Aizhong Ding^a^, Yan Liu^c^, Songqiang Deng^d^, Dayi Zhang^e,*^

^a^College of Water Science, Beijing Normal University, Beijing, PR China

^b^College of Water Resources and Civil Engineering, China Agricultural University, Beijing, PR China

^c^Chinese Research Academy of Environmental Sciences, Beijing, PR China

^d^Research Institute for Environmental Innovation (Suzhou), Tsinghua, Suzhou 215163, PR China

^e^School of Environment, Tsinghua University, Beijing 100084, PR China

*** Corresponding authors:**

Dr. En Xie

College of Water Resources and Civil Engineering, China Agricultural University, Beijing, PR China

Email: [xe@cau.edu.cn](mailto:xe@cau.edu.cn)

Dr. Dayi Zhang

School of Environment, Tsinghua University, Beijing 100084, PR China

Email: zhangdayi@tsinghua.edu.cn

Number of pages: 10

Number of figures: 4

Number of tables: 4

**Measurement of MLSS, TOC, ammonia and orthophosphate**

OD_620_ was used to calculate the MLSS of the activated sludge and was measured by a microplate reader (Synergy 2, Gene Company Ltd, USA). The standard curve was prepared by measuring OD_620_ of the MLSS of the serially diluted activated sludge (1, 10, 50, 100, 150 and 200 times).

TOC standard curve was prepared by measuring known concentrations of potassium hydrogen phthalate (0-1000 mg/L) and mixture of sodium bicarbonate and sodium carbonate (0-100 mg/L) with the same analyzer.

For ammonia measurement, 50-μL of water sample was added into 105 μL of sterile water, following the addition of 10 μL sodium potassium tartrate (500 g/L) and 10 μL Nash reagent in a fresh 96-well bottom-clear microplate. The 100 mL of Nash reagent was prepared by mixing 50 mL of sodium hydroxide (320 g/L), 7.0 g of potassium iodide and 10.0 g of mercuric iodide. After 10-minute color development, the absorbance was measured at 420 nm by a microplate reader (infinite F200 PRO, TECAN, Austria). The standard curve was prepared by measuring known concentrations of NH_4_Cl (0.025-2.000 mg/L) with the above method.

For orthophosphate measurement, 5-μL of water sample was added into 180 μL of sterile water, supplemented with 5 μL of ascorbic acid (100 g/L) and 10 μL of ammonium molybdate-antimony potassium tartrate in a fresh 96-well bottom-clear microplate. The ammonium molybdate-antimony potassium tartrate was prepared by mixing 100 mL of ammonium molybdate (130 g/L), 100 mL of antimony potassium tartrate (3.5 g/L) and 300 mL of sulfuric acid (V/V = 1:1). After 15-minute color development, the absorbance of each well was measured at 700 nm by a microplate reader (Synergy 2, Gene Company Ltd, USA). The standard curve was prepared by measuring known concentrations of KH_2_PO_4_ (0-100 mg/L) with the same protocol as mentioned above.

**AKP activity measurement**

Twenty-five microliters of the activated sludge sample and 25 μL of DiFMUP (200 μM) were added to the same well of a 96-well bottom-clear microplate, and immediately placed in a microplate reader (Synergy 2, Gene Company Ltd, USA). The fluorescent data (excitation at 360 nm, emission at 460 nm) were recorded every 10 minutes for 2 hr. The calibration curve was obtained by diluting acid phosphatase (provided by the kit) to final concentrations of 0, 0.001, 0.005, 0.01, 0.05 and 0.1 U/L, and measured following the same protocol as the activated sludge samples.

**Phosphorus-free M9 medium**

The phosphorus-free M9 medium were prepared as previously reported method (Akhtar and Rehman, 2017) with modification: C_6_H_12_O_6_·H_2_O (5.79 g/L), KCl (3.51 g/L), NH_4_Cl (0.81 g/L), MgSO_4_ (0.98 g/L), FeSO_4_·7H_2_O (0.01 g/L) and MnSO_4_·H_2_O (0.005 g/L), adjusted to pH approximately 7.0 and autoclaved.

**Sequence**

For each sample, 10-digit barcode sequence was added to the 5' end of the forward and reverse primers. The reaction mixture consisted of DNA template (30 ng), 1 μL of each primer (5 μM), 3 μL of BSA (2 ng/μL), 12.5 μL of 2×Taq PCR MasterMix and 7.5 μL of H_2_O. The PCR was performed under the following conditions: 95°C for 5 min; 29 cycles of 95°C for 45 s, 55°C for 50 s and 72°C for 45 s; a final extension at 72°C for 10 min. The amplicons were then subjected to Illumina Miseq PE300 platform for high-throughput sequencing (Allwegene, China). After obtaining the raw reads, low-quality reads with a quality score <25 or a length <200 bp were removed.

**Data analysis**

MLSS (g/L) was calculated according to the calibration curve (*R*^2^ = 0.9991) with the following Equation (1).

$\text{MLSS }\text{= (}{OD}_{620}\text{-0.0875)/0.2923}$ (1)

Quantification of the AKP-associated genes followed the 2^-ΔΔCt^ method. The standard curve was *phoA*=10^(38.111-Ct)/2.982^ (*R*^2^=1.00, copies/g MLSS), *phoD*=10^(56.383-Ct)/6.704^ (*R*^2^=0.98, copies/g MLSS), *phoX*=10^(37.284-Ct)/3.572^ (*R*^2^=0.99, copies/g MLSS) and *phoU*=10^(35.169-Ct)/2.769^ (*R*^2^=1, copies/g MLSS), respectively.


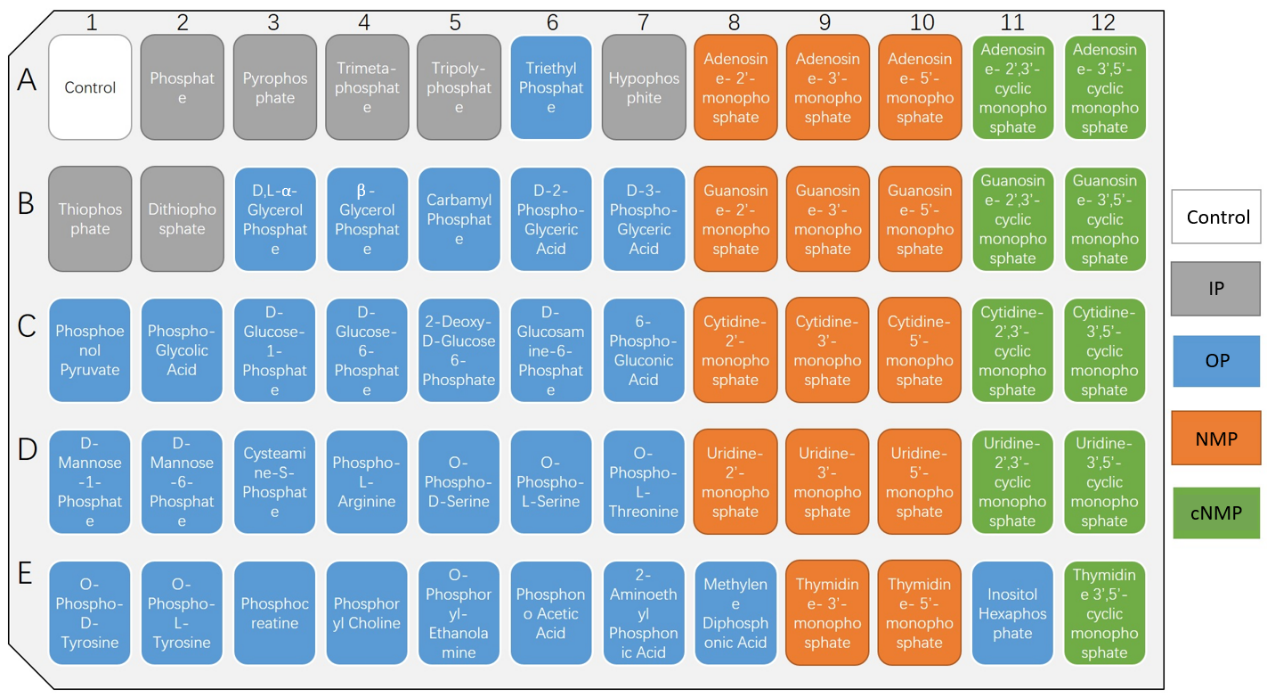


**Figure S1.** Map of BIOLOG PM4 Microplate. White well represents blank; gray well represents inorganic phosphorus (IP); blue well represents other organic phosphorus (OP); brown well represents nucleoside monophosphate (NMP); green well represents cyclic nucleoside monophosphate (cNMP).


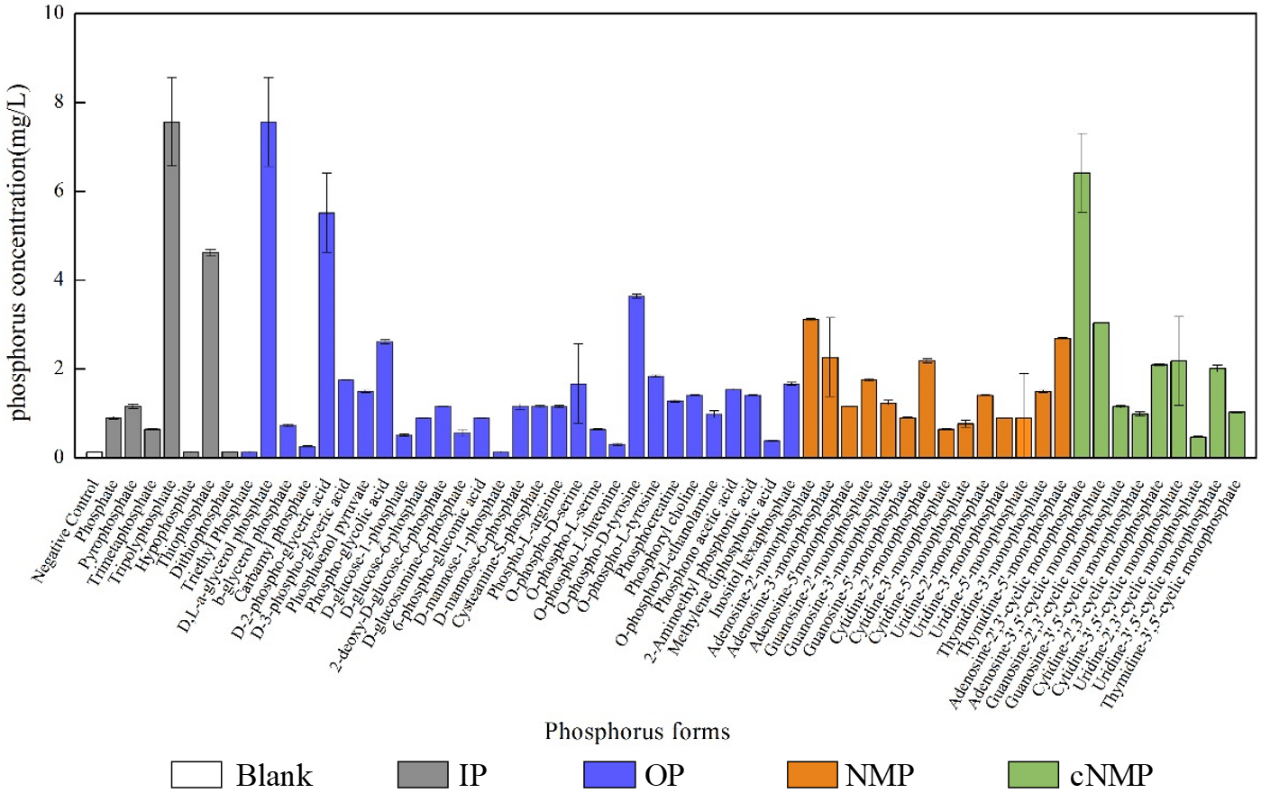

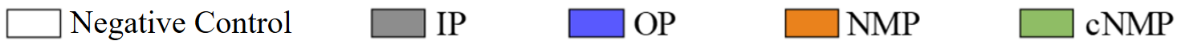


**Figure S2.** Residual orthophosphate concentration in the activated sludge cultivated with different phosphorus sources for 72-hour. White bar represents blank; gray bars represent IP; blue bars represent OP; brown bars represent NMP; green bars represent cNMP.


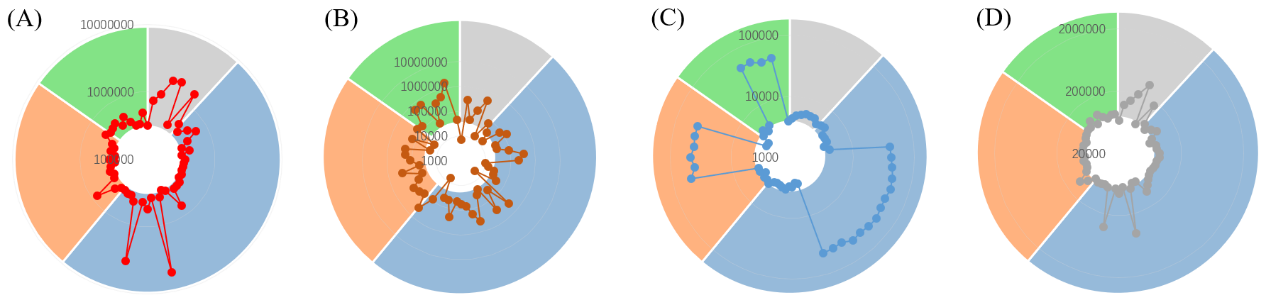


**Figure S3.** The relative abundance of AKP-encoding genes in the activated sludge cultivated with different phosphorus sources. Gray, blue, brown and green background represent IP, OP, NMP and cNMP, respectively.


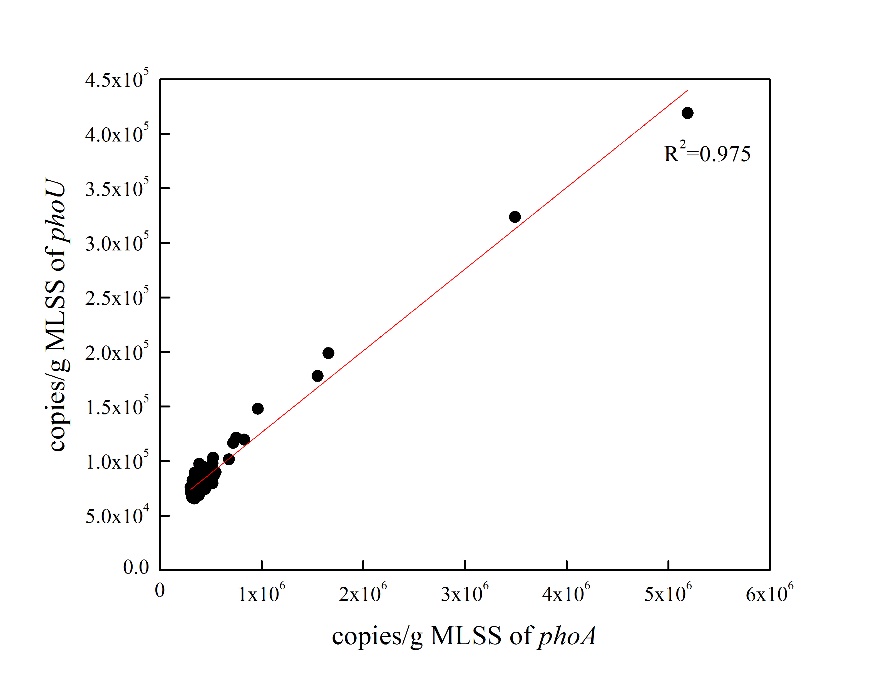
**Figure S4.** The relationship between the relative abundance of *phoA* and *phoU* genes.

**Table S1.** Effluent quality and removal efficiency in SBR.

| **Water quality indices** | **Concentration (mg/L)** | **Removal efficiency** |
| --- | --- | --- |
| **TOC** | 50.46 ± 1.32 | 81.7% |
| **NH_3_-N** | 6.48 ± 0.48 | 86.8% |
| **TN** | 8.82 ± 0.41 | 82.9% |
| **Total phosphorus** | 0.53 ± 0.07 | 89.3% |

**Table S2.** Primers of qPCR for *phoA*, *phoD*, *phoX* and *phoU* genes.

| **Gene** | **Primer sequences (5' to 3')** |
| --- | --- |
| ***phoA*** | CGGACACCAGAAATGCC/AGTGTATTGCCCGGTAAGC |
| ***phoD*** | GACGGCGTGATGNTNTGGACNMG/GTCGTTGGCGACCTCRTGRTCRTC |
| ***phoX*** | CGTCGTTGTAGCCCGTGAT/CCGTTGGAGACATACTTGTAGAGG |
| ***phoU*** | ATCAGTAAAACCATTGCCGG/TCCAGACTTACCAGCAACGG |

**Table S3.** OTU numbers and alpha-diversity indices of the sludge microbial communities cultivated with different phosphorus sources (SD for standard deviation).

| **Phosphorus sources** | **OTUs** | **Chao1** | | **Shannon** | |
| --- | --- | --- | --- | --- | --- |
|  |  | Mean | SD | Mean | SD |
| Negative control | 309 | 313.08 | 18.36 | 3.17 | 0.02 |
| Phosphate | 104 | 136.66 | 21.85 | 1.44 | 0.01 |
| Pyrophosphate | 153 | 195.54 | 27.06 | 2.40 | 0.01 |
| Trimeta-phosphate | 155 | 204.89 | 25.16 | 1.78 | 0.01 |
| Tripoly-phosphate | 93 | 122.20 | 22.53 | 1.26 | 0.01 |
| Hypophosphite | 258 | 292.76 | 12.51 | 3.08 | 0.01 |
| Thiophosphate | 142 | 188.36 | 32.47 | 2.09 | 0.01 |
| Dithiophosphate | 181 | 208.83 | 27.59 | 0.68 | 0.01 |
| Triethyl phosphate | 331 | 350.29 | 25.14 | 3.39 | 0.02 |
| D,L-α-glycerol phosphate | 188 | 220.64 | 28.24 | 2.30 | 0.02 |
| β-glycerol phosphate | 133 | 189.40 | 22.79 | 2.30 | 0.01 |
| Carbamyl phosphate | 233 | 255.72 | 17.27 | 2.72 | 0.01 |
| D-2-phospho-glyceric acid | 121 | 163.61 | 24.60 | 0.24 | 0.01 |
| D-3-phospho-glyceric acid | 119 | 158.97 | 27.26 | 1.12 | 0.01 |
| Phosphoenol pyruvate | 100 | 136.51 | 13.76 | 2.76 | 0.01 |
| Phospho-glycolic acid | 135 | 174.05 | 21.76 | 1.13 | 0.01 |
| D-glucose-1-phosphate | 168 | 193.09 | 22.53 | 3.07 | 0.01 |
| D-glucose-6-phosphate | 92 | 116.24 | 11.54 | 1.77 | 0.01 |
| 2-deoxy-D-glucose-6-phosphate | 97 | 125.01 | 24.44 | 1.02 | 0.01 |
| D-glucosamine-6-phosphate | 147 | 165.28 | 16.65 | 1.72 | 0.01 |
| 6-phospho-glucomic acid | 154 | 168.23 | 26.54 | 0.32 | 0.01 |
| D-mannose-1-phosphate | 232 | 326.83 | 18.31 | 2.32 | 0.01 |
| D-mannose-6-phosphate | 131 | 143.88 | 22.28 | 0.34 | 0.01 |
| Cysteamine-S-phosphate | 118 | 157.25 | 15.83 | 1.61 | 0.01 |
| Phospho-L-arginine | 86 | 168.40 | 21.34 | 1.42 | 0.01 |
| O-phospho-D-serine | 112 | 144.86 | 19.31 | 0.83 | 0.01 |
| O-phospho-L-serine | 263 | 297.84 | 26.97 | 2.55 | 0.02 |
| O-phospho-L-threonine | 168 | 219.90 | 51.50 | 0.33 | 0.01 |
| O-phospho-D-tyrosine | 89 | 113.69 | 31.63 | 1.13 | 0.01 |
| O-phospho-L-tyrosine | 70 | 87.01 | 25.14 | 1.28 | 0.01 |
| Phosphocreatine | 86 | 170.08 | 56.79 | 1.01 | 0.01 |
| Phosphoryl choline | 80 | 79.92 | 11.19 | 2.02 | 0.01 |
| O-phosphoryl-ethanolamine | 42 | 54.63 | 16.53 | 2.44 | 0.01 |
| Phosphono acetic acid | 44 | 63.60 | 7.79 | 1.01 | 0.01 |
| 2-aminoethyl phosphonic acid | 44 | 76.40 | 17.44 | 2.13 | 0.01 |
| Methylene diphosphonic acid | 141 | 158.04 | 7.33 | 2.53 | 0.01 |
| Inositol hexaphosphate | 76 | 99.86 | 17.81 | 2.36 | 0.01 |
| Adenosine-2'-monophosphate | 147 | 203.27 | 27.02 | 1.42 | 0.01 |
| Adenosine-3'-monophosphate | 110 | 156.87 | 24.06 | 1.93 | 0.01 |
| Adenosine-5'monophosphate | 115 | 170.78 | 29.66 | 2.57 | 0.01 |
| Guanosine-2'-monophosphate | 159 | 230.03 | 65.51 | 0.36 | 0.01 |
| Guanosine-3'-monophosphate | 95 | 148.88 | 33.21 | 1.00 | 0.01 |
| Guanosine-5'-monophosphate | 115 | 175.90 | 14.95 | 1.31 | 0.01 |
| Cytidine-2'-monophosphate | 181 | 199.51 | 26.82 | 2.14 | 0.01 |
| Cytidine-3'-monophosphate | 129 | 190.05 | 23.33 | 1.17 | 0.00 |
| Cytidine-5'-monophosphate | 125 | 167.15 | 26.06 | 1.37 | 0.01 |
| Uridine-2'-monophosphate | 163 | 182.56 | 20.45 | 1.73 | 0.01 |
| Uridine-3'-monophosphate | 123 | 211.21 | 27.38 | 2.54 | 0.01 |
| Uridine-5'-monophosphate | 210 | 250.04 | 22.47 | 2.19 | 0.01 |
| Thymidine-3'-monophosphate | 64 | 101.00 | 36.82 | 2.10 | 0.01 |
| Thymidine-5'-monophosphate | 66 | 117.62 | 33.63 | 1.89 | 0.00 |
| Adenosine-2',3'-cyclic monophosphate | 106 | 160.87 | 26.79 | 1.50 | 0.01 |
| Adenosine-3',5'-cyclic monophosphate | 85 | 99.75 | 18.33 | 2.05 | 0.01 |
| Guanosine-2',3'-cyclic monophosphate | 88 | 149.81 | 23.44 | 1.64 | 0.01 |
| Guanosine-3',5'-cyclic monophosphate | 123 | 145.93 | 24.81 | 2.72 | 0.01 |
| Cytidine-2',3'-cyclic monophosphate | 118 | 157.72 | 11.16 | 1.86 | 0.01 |
| Cytidine-3',5'-cyclic monophosphate | 84 | 111.87 | 20.69 | 2.22 | 0.00 |
| Uridine-2',3'-cyclic monophosphate | 135 | 180.52 | 38.30 | 1.58 | 0.01 |
| Uridine-3',5'-cyclic monophosphate | 109 | 142.20 | 28.54 | 2.51 | 0.01 |
| Thymidine-3',5'-cyclic monophosphate | 83 | 91.85 | 13.55 | 3.12 | 0.01 |

**Table S4.** Correlation analyses between MLSS, AKP, residual orthophosphate, TOC removal and ammonia removal.

| **Comparison** | **Correlation** |
| --- | --- |
| **MLSS vs Residual orthophosphate** | r = 0.383** |
| **AKP vs MLSS** | r = 0.364** |
| **AKP vs Residual orthophosphate** | r = 0.261* |
| **R_TOC_ vs Residual orthophosphate** | r = 0.370** |
| **R_TOC_ vs MLSS** | r = 0.560** |
| **R_TOC_ vs AKP** | r = 0.441** |
| **R_TOC_ vs Chao1** | r = -0.383** |
| **R_TOC_ vs Shannon** | r = -0.389** |
| **R_N_ vs Residual orthophosphate** | r = -0.139 |
| **R_N_ vs MLSS** | r = -0.081 |
| **R_N_ vs AKP** | r = -0.023 |
| **R_N_ vs Chao1** | r = -0.024 |
| **R_N_ vs Shannon** | r = -0.161 |

Note: * and ** represent significance at the 0.05 and 0.01 levels, respectively.

R_N_, ammonia removal efficiency

R_TOC_, TOC removal efficiency.
